# Angiogenesis in Chronic Obstructive Pulmonary Disease: A Translational Appraisal

**Published:** 2012-04-30

**Authors:** Alessandro Matarese, Gaetano Santulli

**Affiliations:** 1 University of Naples “Federico II”, Naples, Italy; 2 New York Presbyterian Hospital - Columbia University Medical Center, New York NY, USA

**Keywords:** Angiogenesis, lung, chronic obstructive pulmonary disease

## Abstract

Angiogenesis is a crucial component of lung pathophysiology, not only in cancer but also in other disorders, such as chronic obstructive pulmonary disease (COPD). In COPD angiogenesis is definitely able to control and orchestrate the progression of airway remodeling. Herein, we provide several remarkable translational aspects of angiogenesis in COPD, exploring both basic and clinical research in this field. Indeed, we present a number of pro- and anti-angiogenic factors, which can be also used as potential biomarkers to monitor disease progression.

## INTRODUCTION

Angiogenesis is a complex process that leads to the formation of new blood vessels from a pre-existing vasculature.^1^, ^2^ It is recognized as a key element in both physiological and pathological processes involving neovascularization such as embryogenesis, wound healing, and tumor growth.^3^–^5^ Albeit terminology in this field remains a moot point, we have to clarify the difference between angiogenesis and vasculogenesis (see Table 1).

*Vasculogenesis* is widely used to indicate spontaneous blood-vessel formation, from circulating or tissue-resident angioblasts. This form particularly relates to the embryonic development of the vascular system. *Angiogenesis* denotes the formation of thin-walled endothelium-lined structures with muscular smooth muscle wall and pericytes. This form plays an essential role during the adult life span, also as “repair mechanism” of damaged tissues.

Another difference to point out is between intussusceptive and sprouting angiogenesis. Intussusception is the term for the formation of new blood vessels by the simple splitting of existing ones. This is a fast process, which can take place within hours or even minutes, because it does not need proliferation of endothelial cells. Sprouting (or classic) angiogenesis is the most studied and definitely relies on endothelial cells mitosis and migration. It can be divided into four sequential steps: (1) activation of the endothelial cells which leads to the localized degradation of the basal membrane of the parent vessel and of the extra-cellular surrounding matrix; (2) oriented migration of the endothelial cells in the extracellular matrix; (3) proliferation of the endothelial cells to form sprout and then loops; (4) differentiation of these cells with organization into tubular structures with a new basal lamina. In this way the new capillaries start to form a new vascular network.^3^ Eventually, in the sprouting vessel we can identify two different types of endothelial cells: the tip cells, which are non-proliferative and migrate along vascular endothelial growth factor (VEGF) gradients and the stalk cells, which proliferate in response to VEGF, permitting the vascular sprout to elongate away from the parent vessel.^6^–^8^ Mural cells are recruited to the nascent vessels by a process known as *arteriogenesis*, a term thereby used to designate the formation of medium-sized blood vessels possessing tunica media plus adventitia, especially to bypass arterial stenoses or occlusions.

So far, angiogenesis in the lung has been especially referred to its recognized role in cancer. Angiogenesis is indeed required for tumor growth and metastasis, and it has been shown that high angiogenesis activity is associated with advanced tumor growth, distant metastases, and an adverse prognosis in human malignancies.^1^, ^9^, ^10^ There is also evidence that angiogenesis is a relatively early event during cancer pathogenesis.^9^ Additionally, angiogenic squamous dysplasia, i.e. small lesions where capillary loops project into histologically abnormal bronchial epithelium, has been observed in pre-neoplastic lesions from individuals at high risk of developing lung cancer.^11^ In established invasive tumors, the balance between apoptosis and tumor cell proliferation is dependent on the tumors ability to induce neovascularization to secure oxygen and nutrition for the malignant cells. In the absence of blood vessels, tumor growth is restricted. The mechanism is complex and involves a number of proteins, enzymatic pathways and cytokines, which are able to orchestrate vessel formation, growth pattern, and vascular permeability, modulate host response and affect tumor invasion, metastasis and prognosis.^10^ Transition from the latent to the invasive phase of malignancy is called “angiogenic switch”. During the tumor development, the angiogenic switch is associated with the onset of expression and secretion of angiogenic factors by the tumor cells. Under normal physiological conditions angiogenic mediators establish a balance between the local pro-angiogenic and antiangiogenic functions; angiogenic switch implies a shift in this local balance, and the net balance determines the level of angiogenesis in a tumor.^9^

Beyond the widely recognized role in cancer, angiogenesis is important in other lung disorders that have a lung vascular disease component, such as pulmonary hypertension, chronic obstructive pulmonary disease (COPD) and tuberculosis. In fact, the lung is characterized by double vascularization: the bronchial vasculature, deriving from thoracic aorta (intercostal and mammarian arteries), has a trophic role, while pulmonary system is part of air/blood barrier that plays respiratory function of the lung.^12^ The burden of vasculogenesis and angiogenesis in pneumology can be better understood if one considers that in humans the volume of the lungs increases by more than 20 times during the first 2 years of life. A number of studies in animal models showed that the inhibition of angiogenesis in embryonic lung displays a variety of vascular defects including a significant reduction in formation of air space and capillaries, resulting in distended and under-developed alveoli.^13^, ^14^

Increased angiogenesis occurs in the lungs of patients with pulmonary hypertension, which is driven to a large extent by an exuberant proliferation of endothelial cells.^15^ On the other end of the spectrum we find pulmonary emphysema, with a tissue destruction characterized by a loss of the pulmonary capillary bed.^16^ Indeed, COPD patients have a significantly reduced capillary length and density.^17^

Pulmonary hypertension has in the angiogenic process the most important pathogenetic feature. Other diseases, like COPD, display a loss of tissue and vessels but this aspect does not exclude a role of vasculature in development of disease. We analyze in this review the angiogenetic aspects in COPD, one of the most important lung diseases in term of incidence, prevalence and mortality.

## THE MAIN ACTORS INVOLVED IN THE ANGIOGENIC PROCESS IN THE LUNG

Angiogenesis requires a tightly coordinated guidance from a variety of positive and negative regulators, the balance of which determines the level of ongoing angiogenesis. VEGF is one of the most important players. It is a glycoprotein encoded for by a gene located at chromosome 6 (6p21). There are five forms (A-B-C-D-E) but the most important in angiogenesis is VEGF-A.^18^ This one has six isoforms: VEGF_121_, VEGF_145_, VEGF_165_, VEGF_183_, VEGF_189_, VEGF_206_; all VEGF isoforms are able to bind the receptor tyrosine kinases VEGFR-1 and VEGFR-2. VEGFR-1 binds VEGF with approximately 10-fold higher affinity than VEGFR-2 but, since it has a poor kinase activity, it may act as a silent receptor for VEGF. However, VEGFR-1 is able to enhance VEGF-induced VEGFR-2 signaling during abnormal angiogenesis, because it prevents endothelial cells apoptosis.^19^ VEGF is secreted by endothelial cells, macrophages, stromal cells and malignant cells, but the main target is the endothelium.^1^ The lung represents an organ in which VEGF controls several pathophysiological functions. Indeed, pulmonary tissue contains the highest levels of transcripts among a wide range of organs that express VEGF.^20^ The crucial role of VEGF signaling in lung structure maintenance is supported by the findings that mice treated with an anti VEGFR-2 show respiratory distress and lung prematurity, which can be partially rescued by administration of VEGF.^21^ The function of VEGF in angiogenesis is fundamental; it stimulates the secretion and activation of proteolytic enzymes (matrix metalloproteases, plasminogene activator), and induces degradation of the extra-cellular matrix favoring the proliferation and migration of the endothelial cells and their organization in tubular structures.^22^ In cancer the most important stimulus for the production of VEGF is hypoxia of malignant cells. There is evidence that overexpression of different VEGF isoforms in tumor can induce different clinical, functional and structural characteristics. In particular, VEGF_189_ can induce the dense, small, sprouting microvessels that penetrated deeply from the tumor rim to its core, and has the highest microvessel perfusion and permeability functions.^23^ These characteristics are important in tumorigenesis, systemic metastasis and patient survival in human malignancies including lung cancer. Numerous studies show that a high tumor expression of VEGF_189_ is significantly correlated with large tumors, advanced clinical stage, and systemic metastasis, and represents also an independent prognosis factor in colorectal, renal cell, and non-small-cell lung cancer.^23^ Several lines of evidence indicate that tumor angiogenesis and tumor growth is suppressed when VEGF signal transduction is inhibited.^24^, ^25^ Such inhibition of VEGF signaling is also able to prevent metastases as a result of reduced contact between tumor cells and the capillaries.^26^

Moreover, inhibition or deletion of VEGF is important in bronchopulmonary dysplasia both in animal models and in humans.^27^, ^28^#

Other cytokines partake in angiogenesis, like basic-fibroblast growth factor (B-FGF) and platelet-derived endothelial cell growth factor (PDGF).^4^ Their impact is considered less important then VEGF but especially B-FGF is involved in angiogenesis in lung cancer.^29^ In contrast to VEGF, B-FGF requires basement membrane proteolysis or cell damage for its release and binding to multiple cell targets. Several studies investigated the quantitative evaluation of cytokines as prognostic factor in lung cancer. Some studies used immunohistochemical methods both for VEGF and B-FGF, but presented a great variability in outcome. This method is thereby not applicable in clinical practice. The serum dosage of VEGF or B-FGF is simple and can be serially repeated. However, also in this case there are controversial results, probably due to different methods and a not clear cut-off for “normal” values, especially for VEGF. Other limitations concern that its level is linked to other variables (platelet count, white body cells, performance status, tumor volume etc.). B-FGF serum dosage has the same limitations but there is more evidence for a correlation with clinical outcome. Thus, it will be potentially more useful in practice.^30^

Another factor whose importance for angiogenesis in the lung has recently emerged is vascular endothelial statin (VE-statin), better known as epidermal growth factor-like domain 7 (EGFL7). EGFL7 is a secreted protein that is expressed by and acts on endothelial cells. Its function in angiogenesis is, at least in part, mediated by modulating Notch signaling.^31^ Of interest, in vertebrates the *egfl7* gene encodes within intron 7 the biologically active micro-RNAs miR-126 and mir-126*, which are relevant for the development of the cardiovascular system.

The ultimate goal of translational research is to help the discovery of appropriate therapies and aid in patient management.^32^–^38^ Because of the importance of VEGF in angiogenesis and its role in cancer, this cytokine is a good target for therapy. To date, only bevacizumab, a monoclonal antibody against VEGF, has proven to be an effective agent when combined with chemotherapy in advanced lung cancer. Sandler and colleagues validated bevacizumab for lung cancer therapy and showed a clear and meaningful survival advantage with bevacizumab plus chemotherapy vs. chemotherapy alone.^39^ There are other VEGF specific antibodies under validation, the most important of them is sunitinib, which has shown a great response rate as single agent and appears to be more useful than bevacizumab. Unfortunately, these agents showed a significant toxicity: hypertension. This is a class-related effect of VEGF inhibitors, but management with oral antihypertensive drugs is usually direct and effective; increased vascular events, both arterial and venous, have been seen with bevacizumab in patients with colon cancer and need to be considered in patients with lung cancer. Bleeding in the form of hemoptysis is the most worrisome toxicity seen with bevacizumab in patients with lung cancer.^40^ Current National Comprehensive Cancer Network (NCCN) Guidelines recommend bevacizumab in conjunction with chemotherapy in patients with Eastern Cooperative Oncology Group (ECOG) performance status (from 0 to 5, with 0 denoting perfect health and 5 death)^41^ 0–2 who meet the following eligibility criteria: nonsquamous cell histology and no hemoptysis, central nervous system metastasis, and ongoing therapeutic anticoagulation.^42^ The same guidelines also states that any regimen with a high risk of thrombocytopenia, and therefore risk of bleeding, should be used with caution.^42^

## COPD AND ANGIOGENESIS

COPD is characterized by a persistent airflow limitation and a remodeling of small airways, mainly due to an abnormal inflammatory response to cigarette smoking and outdoor air pollution.^43^ COPD is associated with substantial burden in terms of prevalence of disease, death and disability risk, as well as health care costs. Research in the past two decades revealed pathological features of lung tissue remodeling in COPD patients: changes in mucosal tissue, fiber types and/or fibrosis, pulmonary and systemic inflammation, lung vascular remodeling, and angiogenesis.^43^ Inflammation is a pivotal pathological feature of COPD and may promote angiogenesis through means an influx of inflammatory cells (neutrophils, macrophages and CD8+ T lymphocytes) in the lumen and the wall of the bronchial and bronchiolar airways and parenchyma.^44^

Furthermore, inflammation can induce the production of angiogenetic mediators, such as tumor necrosis factor (TNF)-alpha, which displays a widely recognized angiogenic activity.^1^, ^45^ Also, inflammatory tissue is often hypoxic, and hypoxia may induce angiogenesis through the upregulation of pro-angiogenic factors such as the above mentioned VEGF or B-FGF.^1^, ^46^ On this ground, Kranenburg and colleagues showed that COPD is associated with an increased expression of VEGF in the bronchial, bronchiolar, and alveolar epithelium and in bronchiolar macrophages, as well as airway smooth muscle and vascular smooth muscle cells in both the bronchiolar and alveolar regions.^47^ The same Authors postulated that VEGF and its receptor system might contribute to the maintenance of endothelial and epithelial cell viability in response to injury. Other studies have noticed the involvement of bronchial vasculature in the airway remodeling occurring in smokers with COPD and normal lung function, which displayed an increase in bronchial vascularity, expressed in terms of both number of vessels and vascular area, compared to healthy non-smokers. These features are associated with increase expression of integrin α_v_β_3_ and VEGF.^7^ All these lines of evidence indicate that angiogenesis partakes in remodeling of airways in COPD probably already in preclinical stage as part of the inflammatory response to smoking.

COPD is associated with vascular remodeling that changes the pulmonary circulation. Hypoxia has been classically considered the major pathogenic mechanism of these changes. Some studies suggest that the natural history of this vascular remodeling in COPD might commence at moderate degrees of disease severity.^48^ Other recent observations indicate that muscular and bronchiolar arteries have increased adventitial infiltration of CD8^+^ T lymphocytes. Besides, their intimal thickening is correlated with the amount of total collagen deposition.^49^

VEGF plays an important role in this context: its expression is increased in pulmonary muscular arteries of patients with moderate COPD and also in smokers with normal lung function, as compared with non-smokers, and this expression is associated with the enlargement of the arterial wall. The response of pulmonary vasculature to hypoxia relies on the presence of vascular progenitor cells that are present on the endothelial surface and the intimal space of pulmonary arteries of COPD patients. The number of these cells was associated with the response to hypoxic stimulus and with the enlargement of the arterial wall, too.^50^

In contrast, in patients with severe emphysema the immunohistochemical expression of VEGF in pulmonary arteries, such as its protein content in lung tissue, tends to be low, despite intense vascular remodeling.^51^ Moreover, murine models in which lung VEGF was deleted by means of Cre/Lox technique^52^ show emphysema after four weeks of intratracheal instillation of adenoassociated virus Cre.^53^ Other experiments showed that chronic cigarette smoking and administration of a VEGF receptor blocker caused lung cell apoptosis and significant airspace enlargement.^54^ Some recent hypotheses suggest that lung endothelial cells are particularly VEGF-dependent for their survival. Apoptosis of endothelial cells leading to the loss of capillaries may thereby be a central mechanism in patients with emphysema. There is growing evidence that this mechanism is more important than the classic protease/antiprotease imbalance hypothesis for lung destruction induced by cigarette smoking.

Because endothelium plays a key role in regulating cell growth in vessel wall, it has been hypothesized that endothelial dysfunction might be an initiating event that promotes vessel remodeling in COPD. Endothelial monocyte-activating protein 2 (EMAPII) is a pro-inflammatory endothelial- and monocyte-activating polypeptide and an anti-angiogenic molecule, which specifically induces apoptosis in endothelial cells.^55^ This property of EMAPII may be highly relevant to emphysema because endothelial cell apoptosis is sufficient to recapitulate key pathological features of this disease.^55^ EMAPII is expressed in the cytosol of all cell types and is upregulated by general cellular stress, hypoxia, and LPS. High EMAPII levels in the bronchoalveolar lavage fluid (BALF) and lung parenchyma of individuals with COPD persist even after smoking has been ceased. Also, targeting the proapoptotic protein EMAPII via antibody neutralization significantly reduced the development of cigarette-smoke-induced emphysema despite prior, concurrent, and subsequent exposure of the animals to cigarette-smoke, suggesting EMAPII could be a therapeutic target in this kind of chronic obstructive lung disease.^56^ Summing up, the pathobiology of angiogenesis and vascular remodeling in COPD is still not fully understood.

## CONCLUSION

Angiogenesis is a central component of lung pathophysiology, not only in cancer but also in other chronic diseases, such as COPD, in which it controls the progression of airway remodeling. Several remarkable translational aspects arise from our review. Different pro- and anti-angiogenetic factors^3^, ^7^, ^24^, ^25^ can be used as potential biomarkers to monitor disease progression by measuring their blood or BALF concentrations. Of course, large-scale studies are warranted to find the best suitable marker.

## Figures and Tables

**Table 1:** 

**Angiogenesis**	Budding of new capillary branches from existing blood vessels
**Vasculogenesis**	*De novo* blood vessel formation during embryogenesis
**Arteriogenesis**	Adaptive outgrowth of pre-existent collateral arteries in response to hemodynamic changes
